# Glibenclamide inhibits BK polyomavirus infection in kidney cells through CFTR blockade

**DOI:** 10.1016/j.antiviral.2020.104778

**Published:** 2020-06

**Authors:** Margarita-Maria Panou, Michelle Antoni, Ethan L. Morgan, Eleni-Anna Loundras, Christopher W. Wasson, Matthew Welberry-Smith, Jamel Mankouri, Andrew Macdonald

**Affiliations:** a School of Molecular and Cellular Biology, Faculty of Biological Sciences and Astbury Centre for Structural Molecular Biology, University of Leeds, Leeds, West Yorkshire, LS2 9JT, United Kingdom; b St-James University Hospital, Leeds, West Yorkshire, LS9 7TF, United Kingdom

**Keywords:** PVAN, BK virus, Ion channel, Glibenclamide, CFTR

## Abstract

BK polyomavirus (BKPyV) is a ubiquitous pathogen in the human population that is asymptomatic in healthy individuals, but can be life-threatening in those undergoing kidney transplant. To-date, no vaccines or anti-viral therapies are available to treat human BKPyV infections. New therapeutic strategies are urgently required. In this study, using a rational pharmacological screening regimen of known ion channel modulating compounds, we show that BKPyV requires cystic fibrosis transmembrane conductance regulator (CFTR) activity to infect primary renal proximal tubular epithelial cells. Disrupting CFTR function through treatment with the clinically available drug glibenclamide, the CFTR inhibitor CFTR_172_, or CFTR-silencing, all reduced BKPyV infection. Specifically, time of addition assays and the assessment of the exposure of VP2/VP3 minor capsid proteins indicated a role for CFTR during BKPyV transport to the endoplasmic reticulum, an essential step during the early stages of BKPyV infection. We thus establish CFTR as an important host-factor in the BKPyV life cycle and reveal CFTR modulators as potential anti-BKPyV therapies.

## Highlights

• BK polyomavirus (BKPyV) is life-threatening in those undergoing kidney transplant.
• BKPyV requires CFTR to infect primary kidney cells.
• Disrupting CFTR function pharmacologically reduces BKPyV infection.
• CFTR is required during BKPyV transport to the endoplasmic reticulum.

## 1 Introduction

BK polyomavirus (BKPyV) was first isolated in 1971 and is a significant risk factor for renal transplant dysfunction and allograft loss (Chong et al., 2019). BKPyV was identified at a similar time to the related JC polyomavirus (JCPyV) (Gardner et al., 1971), which remained the only human polyomaviruses identified until 2007. To date, 13 human polyomaviruses have been discovered (Moens et al., 2017) with interest in this virus family remerging due to their increasing association with human disease (DeCaprio and Garcea, 2013; Stakaityte et al., 2014; Helle et al., 2017).

BKPyV establishes a life-long persistent infection of the urinary tract and kidneys in >80% of adults (Kean et al., 2009; Chesters et al., 1983). BKPyV causes a sub-clinical infection in healthy individuals, but a number of diseases are associated with uncontrolled virus replication in the immunosuppressed. Polyomavirus-associated haemorrhagic cystitis (PVHC) afflicts 10–25% of bone marrow transplant recipients and polyomavirus-associated nephropathy (PVAN) occurs in up to 10% of kidney transplant patients, leading to the lytic destruction of tubular epithelial cells and a loss of graft function in up to 80% of cases (Helle et al., 2017; Ahsan and Shah, 2006). The treatment of BKPyV-associated disease is limited to immune suppression medication to allow the host immune response to fight the infection, coupled to use of broad-spectrum nucleoside analogues. However, the reduction of immunosuppression enhances the risk of host graft rejection whilst anti-viral nucleoside analogues display questionable efficacy and known nephrotoxicity (Kuypers, 2012). JCPyV can, on rare occasions also cause PVAN. A clear and unmet need to study this family of viruses and identify potent and direct anti-viral therapeutics therefore exists.

BKPyV consists of a capsid containing a ~5 kb circular double-stranded DNA genome (Bennett et al., 2012). The genome is composed of two highly conserved regions that code for early and late viral proteins, separated by a non-coding control region containing the origin of DNA replication and promoter region. The early region encodes the large and small tumour antigens, necessary for regulating virus transcription and replication. The late region encodes the major capsid protein VP1 and the minor capsid proteins VP2 and VP3 (Helle et al., 2017). BKPyV virions consist of 360 copies of VP1 that form 72 pentamers arranged in an icosahedral capsid (Hurdiss et al., 2016). Each VP1 monomer interacts with a neighbouring monomer through a C-terminal extending arm, and through hydrophobic interactions with internal VP2 and VP3 molecules (Hurdiss et al., 2016, 2018). Within the late region, BKPyV also encodes a small hydrophobic auxiliary protein termed the agnoprotein, which is essential for virus release from infected cells (Gerits and Moens, 2012; Panou et al., 2018).

BKPyV infects the epithelial lining of the collective ducts, the transitional epithelial cells of the renal calyces, the parietal epithelium of the Bowman's capsule, and the transitional epithelium of the renal pelvis and urinary tract (Meehan et al., 2006). The major function of these epithelial tissues is to maintain ion, solute, and water homeostasis through a concerted effort of ion transporters that control sodium (Na^+^) and potassium (K^+^) reabsorption, K^+^ efflux, and chloride (Cl^−^) secretion (Palmer and Sackin, 1988). At the cellular level, ion channels regulate many important physiological functions including cell volume, apoptosis, and the ionic homeostasis of intracellular vesicles/organelles (Bardou et al., 2009; Roger et al., 2015; O'Grady and Lee, 2003). These cellular functions frequently overlap with those hijacked during virus infection. Accordingly, the pharmacological modulation of host cell ion channel activity can impede an array of important human viruses including Ebola virus, hepatitis C virus and bunyaviruses (Charlton et al., 2019, Hover et al., 2017, 2018; Igloi et al., 2015; Sakurai et al., 2015). However, a requirement for ionic homeostasis and its contribution to the ability of BKPyV to persistently infect cells has not been explored.

In this study, using an established panel of ion channel modulating drugs, we assessed the contribution of cellular ion channel activity to the BKPyV lifecycle. We demonstrate that pharmacological blockers of the cystic fibrosis transmembrane conductance regulator (CFTR) impede BKPyV infection in primary kidney cells. One of these blockers, glibenclamide, is a clinically approved anti-diabetic drug, thus revealing a potent FDA approved anti-BKPyV therapeutic. We finally show that the CFTR blockers inhibit the early stages of BKPyV infection following virus penetration into cells. This furthers our understanding of BKPyV infection and replication strategies and reveals new and exciting strategies for much needed anti-BKPyV therapeutics.

## 2 Results

### 2.1 Glibenclamide inhibits BKPyV infection

**Fig. 1:**
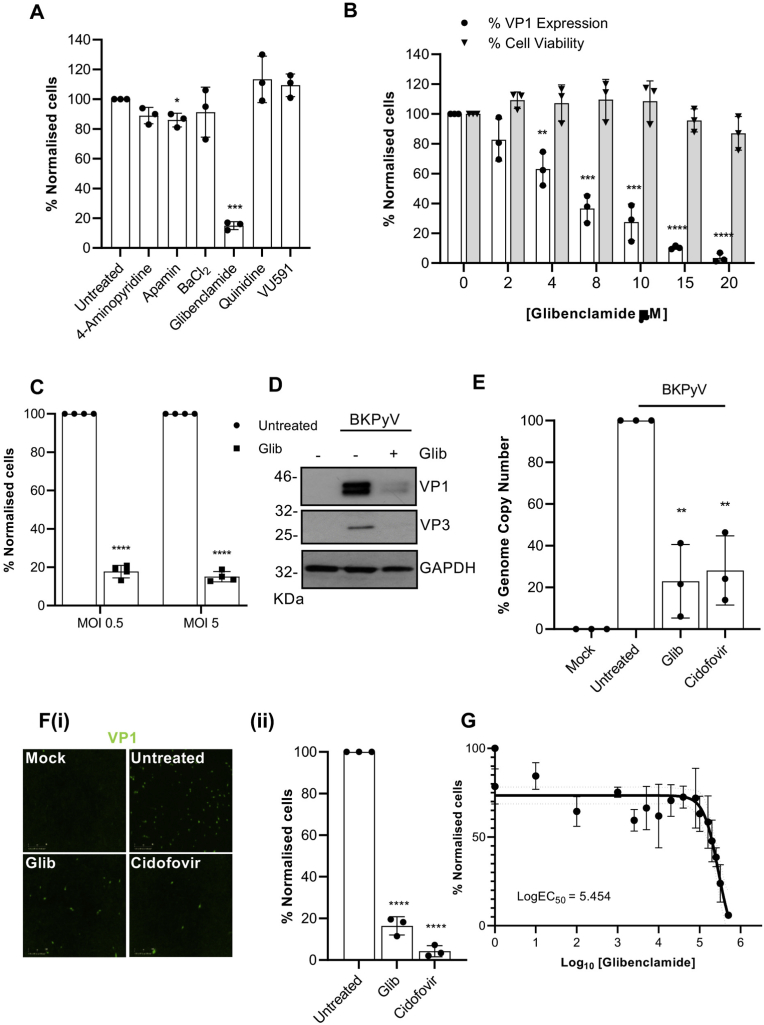
**K**^**+**^**channel inhibition impairs BKPyV infection. A**) BKPyV infected RPTE cells were treated with 4-AP (0.5 mM), apamin (0.5 μM), BaCl_2_ (1 mM), glibenclamide (20 μM), quinidine (50 μM), TEA (20 mM) and VU591 (10 μM) for 48 h. Cells were fixed and probed with anti-VP1 and anti-mouse Alexa-fluor 488 secondary antibodies. The percentage of BKPyV infected cells was quantified using IncuCyte ZOOM software and normalised to untreated cells. **B)** BKPyV infected RPTE cells were treated with increasing concentrations (0–20 μM) of glibenclamide. At 48 hpi, cells were fixed and stained for BKPyV VP1. The percentage of BKPyV infected cells was normalised to untreated cells (open bars). Cell viability was assessed by MTT assays. Values were normalised to untreated controls (grey bars). **C)** RPTE cells were infected with BKPyV at MOI 0.5 or 5 and treated with 20 μM glibenclamide. The percentage of BKPyV infected cells was quantified using IncuCyte ZOOM software and normalised to untreated cells. **D)** Lysates from BKPyV infected RPTE cells treated with glibenclamide (20 μM) were resolved by SDS-PAGE and probed with anti-VP1, anti-VP2/VP3, and anti-GAPDH antibodies. Representative western blots are shown. **E)** DNA was extracted from BKPyV infected RPTE cells treated with glibenclamide (20 μM) or cidofovir (15 mg/ml) and BKPyV genome replication was determined by qPCR analysis. Levels were normalised to untreated controls. **Fi)** BKPyV infected RPTE cells were treated with glibenclamide (20 μM) or cidofovir (15 mg/ml) and the media fraction was harvested and used to infect naïve RPTE cells. **Fii)** The percentage of BKPyV infected cells was quantified using IncuCyte ZOOM software and normalised to untreated cells. **G)** BKPyV infected RPTE cells were treated with a range of concentrations of glibenclamide and concentration response curves were constructed. Data show mean values with SD (*n* = 3); data in Fig. 1A were compared using Welch's test, Fig. 1B was analysed by 2-way ANOVA and a two-tailed unpaired *t*-test was used for the remaining figures (*P < 0.05, **P < 0.005, ***P < 0.0005, ****P < 0.0001).

Ion channels have emerged as essential host factors in the lifecycles of a number of important human viruses (Hover et al., 2017). To determine if the activity of kidney-expressed channels are required during the BKPyV lifecycle, BKPyV infection assays were performed in the presence of well-characterised modulators of renal K^+^ channels. We initially selected K^+^ channels as they represent the largest ion channel family (over 70 different K^+^ channel genes per cell) for which an array of blockers are available (Hover et al., 2017). Assays were performed in primary renal proximal tubular epithelial (RPTE) cells, used as a physiologically relevant cell culture infection model that maintains the apical and basolateral membrane domains critical for ion transport (Low et al., 2004). BKPyV infection was measured through the expression of the VP1 major capsid protein; known to correlate with virus production (Panou et al., 2018; Low et al., 2004). Drugs at pharmacologically relevant concentrations were added to cells with virus supernatants, permitting the inhibition of all lifecycle stages. Data from these assays revealed that inhibitors of voltage gated K^+^ channels (4-aminopyridine, 4-AP), calcium-activated K^+^ channels (Apamin), inwardly rectifying K^+^ channels (BaCl_2_), two-pore K^+^ channels (quinidine) and the renal outer medullary K^+^ channel (ROMK, VU591) did not influence BKPyV infection, whilst the K_ATP_ channel inhibitor glibenclamide produced ≥ 80% inhibition of BKPyV infection (Fig. 1A).

To further explore the inhibitory effects of glibenclamide, infection assays were performed at a range of glibenclamide concentrations (0–20 μM). We observed a concentration-dependent decrease in VP1 levels (Fig. 1B, *open bars*) with a negligible impact on RPTE cell viability (≥80% at all concentrations assessed; Fig. 1B, *grey bars*). The inhibition of BKPyV occurred irrespective of the multiplicity of infection of BKPyV, as a similar impairment was observed at an MOI of 0.5 and 5 (≥80% decrease; p ≤ 0.0001) in the presence of 20 μM glibenclamide (Fig. 1C). Glibenclamide also inhibited the expression of the minor capsid protein VP3, confirming that the effects were not restricted to VP1 (Fig. 1D). Glibenclamide also reduced viral genome production as confirmed through qPCR analysis of BKPyV genome copy number (≥80% decrease; p ≤ 0.005 Fig. 1E). The levels of inhibition were comparable to cidofovir; a known inhibitor of BKPyV replication (Fig. 1E) (Safrin et al., 1997). We further assessed the impact of glibenclamide and cidofovir treatment on the production of infectious BKPyV progeny by infecting naïve RPTE cells with the media from virus infected control and inhibitor treated cells, followed by staining for the production of the VP1 capsid protein. Consistent with these findings, we observed a ≥80% (p ≤ 0.0001) loss of infectious virus production in cells treated with either glibenclamide or cidofovir (Fig. 1Fi-ii). The EC_50_ of glibenclamide inhibition against BKPyV was found to be 5.454 μM (Fig. 1G). Taken together, these data identified a requirement for glibenclamide sensitive channels during the BKPyV lifecycle in primary RPTE cells.

### 2.2 K_ATP_ channels are not required for BKPyV infection

Glibenclamide is commonly used to treat type II neonatal diabetes through the pharmacological blockade of K_ATP_ channels (Bhattia et al., 1970; Mikhailov et al., 2001; Gudat et al., 1998). The closure of K_ATP_ channels results in a loss of K^+^ efflux and membrane depolarization (Nichols and Lederer, 1991; Nichols et al., 1991; Aguilar-Bryan et al., 1992; Niki et al., 1989). K_ATP_ channels are composed of an inwardly rectifying potassium channel K_IR_ (K_IR_6.1, K_IR_6.2) and a sulphonylurea sub-unit (SUR1, SUR2A and SUR2B) which regulate the activity of K_IR_ through their sensitivity to the ATP/ADP ratio and other metabolites (Larsson et al., 1993; Hilgemann and Ball, 1996; Challinor-Rogers and McPherson, 1994; Glukhov et al., 2013; Ruknudin et al., 1998). Specifically, glibenclamide binds to SUR1 to inhibit K_ATP_ channel activity (Mikhailov et al., 2001). Whilst the potent anti-BKPyV activity of glibenclamide implicates this channel family as required during BKPyV infection, the insensitivity of BKPyV to BaCl_2_ questions K_ATP_ involvement, since BaCl_2_ is a known inhibitor of K_IR_ subunits (Wellman et al., 1996; Teramoto et al., 2006; Lippiat et al., 2002). We therefore investigated in further detail if K_ATP_ channels are the cellular target influencing BKPyV infection.

**Fig. 2:**
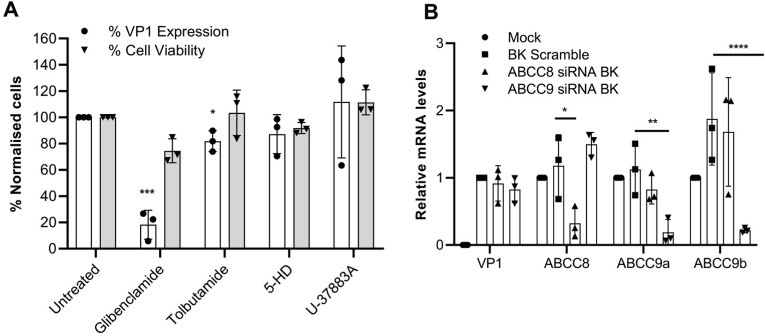
**Glibenclamide inhibits BKPyV independently of K**_**ATP**_**channels. A)** BKPyV infected RPTE cells were treated with glibenclamide (20 μM), tolbutamide (150 μM), 5-HD (500 mM) and U-37883A (50 μM). At 48 hpi cells were fixed and stained for BKPyV VP1. Widefield images were captured using an IncuCyte ZOOM. The percentage of BKPyV infected cells was quantified using IncuCyte ZOOM software and normalised to untreated cells (Open bars). Cell viability was assessed by MTT assays. Values were normalised to untreated controls (grey bars). **B)** BK-VP1 expression is unaffected by SUR1 (ABCC8) and SUR2A/B (ABCC9a/b) silencing. Cells were infected with BKPyV following the siRNA-mediated silencing of SUR1, SUR2A and SUR2B. Data are the mean ± SD normalised to scrambled RNA controls. Data were compared using a 2-way ANOVA (*P < 0.05, **P < 0.005, ***P < 0.0005, ****P < 0.0001).

Firstly, we assessed other characterised K_ATP_ blocking drugs for their effects on BKPyV. Tolbutamide, a high-affinity K_ATP_ channel blocker; guanidine; 4-morpholinecarboximidine-N-1-adamantyl-N′-cyclohexyl hydrochloride (U-37883A), a sub-micromolar K_ATP_ blocker; and 5-hydroxydecanoate (5-HD), a mitochondria K_ATP_ channel specific inhibitor were investigated at concentrations known to inhibit K_ATP_ function (Katsumata and Katsumata, 1990; Bijlstra et al., 1996; Meisheri et al., 1993; Teramoto, 2006; Nakagawa et al., 2005; Sarre et al., 2005). Analysis of the percentage of BKPyV infected cells revealed a modest but significant reduction in BKPyV infection in the presence of tolbutamide, whilst no effects of U-37883A or 5-HD on BKPyV were observed (glibenclamide ≥80% decrease; p < 0.0005 *vs.* tolbutamide 20% decrease, p < 0.05) (Fig. 2A). These data suggested that other K_ATP_ channel inhibitors fail to recapitulate the inhibition by glibenclamide.

We further investigated the involvement of K_ATP_ channels by silencing the expression of SUR1, SUR2A and SUR2B in RPTE cells prior to BKPyV infection (Fig. 2B). qRT‐PCR data demonstrated no loss of VP1 expression, and thus BKPyV infection in the face of successful *ABCC8* (SUR1), *ABCC9A* (SUR2A) and *ABCC9B* (SUR2B) silencing (Fig. 2B). Taken together, these data strongly suggest that the inhibitory effects of glibenclamide on BKPyV infection are independent of K_ATP_ channels.

### 2.3 CFTR is required during BKPyV infection

**Fig. 3:**
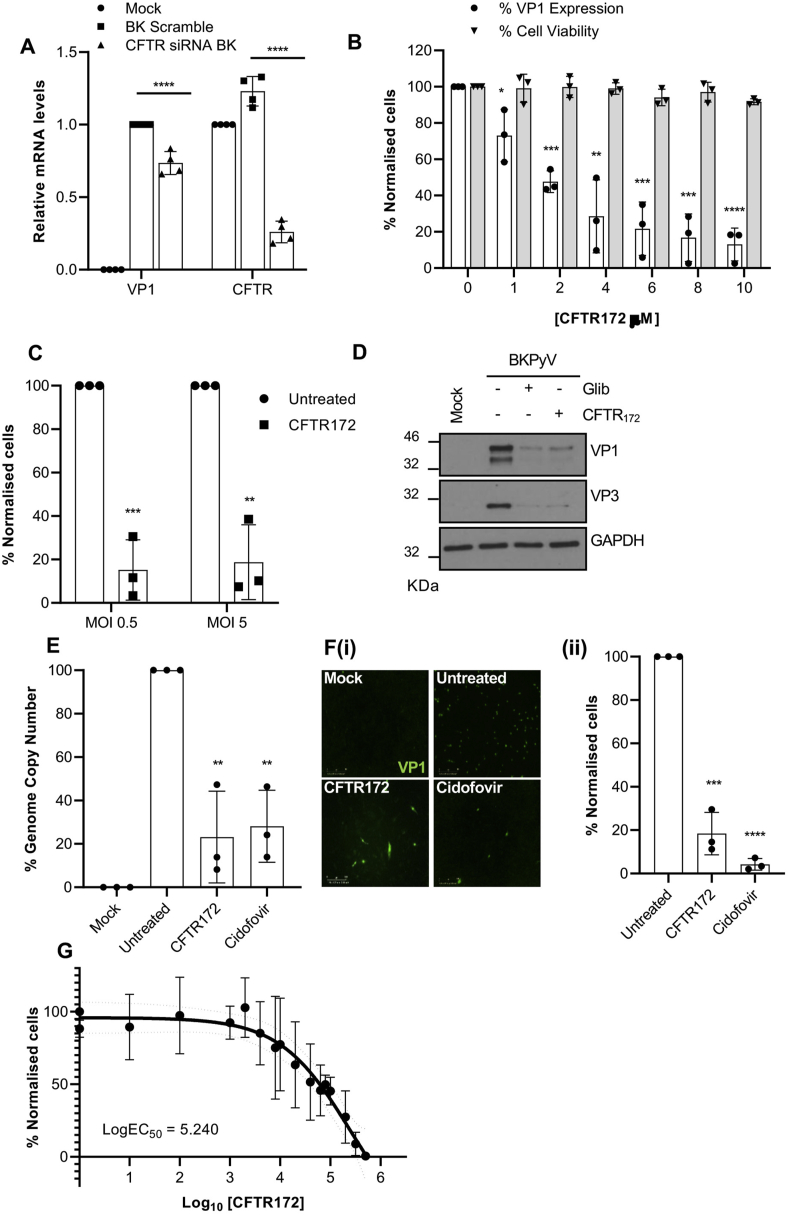
**Pharmacological inhibition of CFTR impedes BKPyV infection. A)** Cells were infected with BKPyV following the siRNA-mediated silencing of CFTR. Data for Fig. 3A are the mean ± SD normalised to scrambled RNA controls. **B)** BKPyV infected RPTE cells were treated with increasing concentrations (0–10 μM) of CFTR_172_. At 48 hpi, cells were fixed and stained for BKPyV VP1. The percentage of BKPyV infected cells was quantified using IncuCyte ZOOM software and normalised to untreated cells (Open bars). Cell viability was assessed by MTT assays (grey bars). **C)** RPTE cells were infected with BKPyV at an MOI of 0.5 or 5 and treated with 10 μM of CFTR_172_. The percentage of BKPyV infected cells was quantified using IncuCyte ZOOM software and normalised to untreated cells. **D)** RPTE cells were treated with CFTR_172_ (10 μM) or glibenclamide (20 μM) for the first 10 h of BKPyV infection. Cells were then washed and the media was replaced with growth media. Lysates were generated at 48 hpi and analysed by SDS PAGE. Samples were probed with anti-VP1 (ab597) and anti-VP2/VP3 (ab53983) antibodies. GAPDH served as a loading control. **E)** DNA was extracted from BKPyV infected RPTE cells treated with CFTR_172_ or cidofovir and BKPyV genome replication was determined by qPCR analysis. Expression levels were normalised to untreated controls. **Fi)** BKPyV infected RPTE cells were treated with CFTR_172_ (10 μM) or cidofovir (15 mg/ml) and the media fraction was harvested and used to infect naïve RPTE cells. **Fii)** The percentage of BKPyV infected cells was quantified using IncuCyte ZOOM software and normalised to untreated cells. **G)** BKPyV infected RPTE cells were treated with a range of concentrations of CFTR_172_ and concentration response curves were constructed. Data are the mean ± SD; data were compared using a two-tailed unpaired *t*-test unless stated otherwise. Data for Fig. 3A were compared using a 2-way ANOVA, Fig. 3B were compared using a 2-way ANOVA, (*P < 0.05, **P < 0.005, ***P < 0.0005, ****P < 0.0001).

In addition to K_ATP_ channels, glibenclamide is a known blocker of the cystic fibrosis transmembrane conductance regulator (CFTR) (Sheppard and Welsh, 1992; Sheppard and Robinson, 1997). CFTR is an ABC transporter that is also a Cl^−^ permeable channel expressed in all nephron segments and the principal cells of the cortical and medullary collecting ducts (Souza-Menezes and Morales, 2009). A potential role for CFTR in BKPyV infection was first investigated through CFTR siRNA silencing experiments. The transfection of RPTE cells with CFTR specific siRNA yielded a ~75% knockdown in *CFTR* mRNA expression (p ≤ 0.0001), which resulted in a ~25% decrease in VP1 expression (p ≤ 0.0001) compared to scrambled siRNA controls (Fig. 3A). Upon infection with BKPyV, we also observed a ~25% (p ≤ 0.0003) increase in the levels of *CFTR* mRNA expression, suggesting that the virus may up-regulate *CFTR* expression. A challenge with these experiments was that the biochemical half-life of CFTR exceeds 48 h compared to the ~25.5 h reported for SUR1, SUR2A and SUR2B. We therefore reasoned that a pharmacological approach to CFTR inhibition was more suitable. To achieve this, BKPyV infections were performed in the presence of the CFTR specific inhibitor CFTR_172_ (Caci et al., 2008). In these assays, concentrations of CFTR_172_ as low as 10 μM significantly inhibited VP1 expression (Fig. 3B) (≥80% decrease at 10 μm; p ≤ 0.0001, *open bars*), with minimal impact on RPTE cell viability (Fig. 3B, *grey bars*). As observed with glibenclamide, the inhibition of BKPyV occurred at MOIs of 0.5 and 5 and so was independent of BKPyV MOI (Fig. 3C ≥ 80% decrease; p ≤ 0.005) and reduced VP1 and VP3 protein expression (Fig. 3D). BKPyV genome copy numbers were also reduced upon treatment with CFTR_172_ to levels comparable to cidofovir (Fig. 3E 80% decrease; p < 0.005), which correlated with a significant impairment in virus replication, as judged by VP1 and VP3 protein expression (Fig. 3D). Importantly, CFTR_172_ treatment also reduced the production of infectious progeny virus from RPTE cells (Fig. 3Fi-ii, CFTR_172_ ≥80% decrease; p ≤ 0.0005, cidofovir ≥90% decrease; p ≤ 0.0001). The EC_50_ of CFTR against BKPyV was 5.24 μM (Fig. 3G). The combination of our CFTR_172,_ glibenclamide and CFTR depletion experiments therefore support a role for kidneyexpressed CFTR as an important host factor during BKPyV infection.

### 2.4 CFTR is required during the early stages of BKPyV infection

**Fig. 4:**
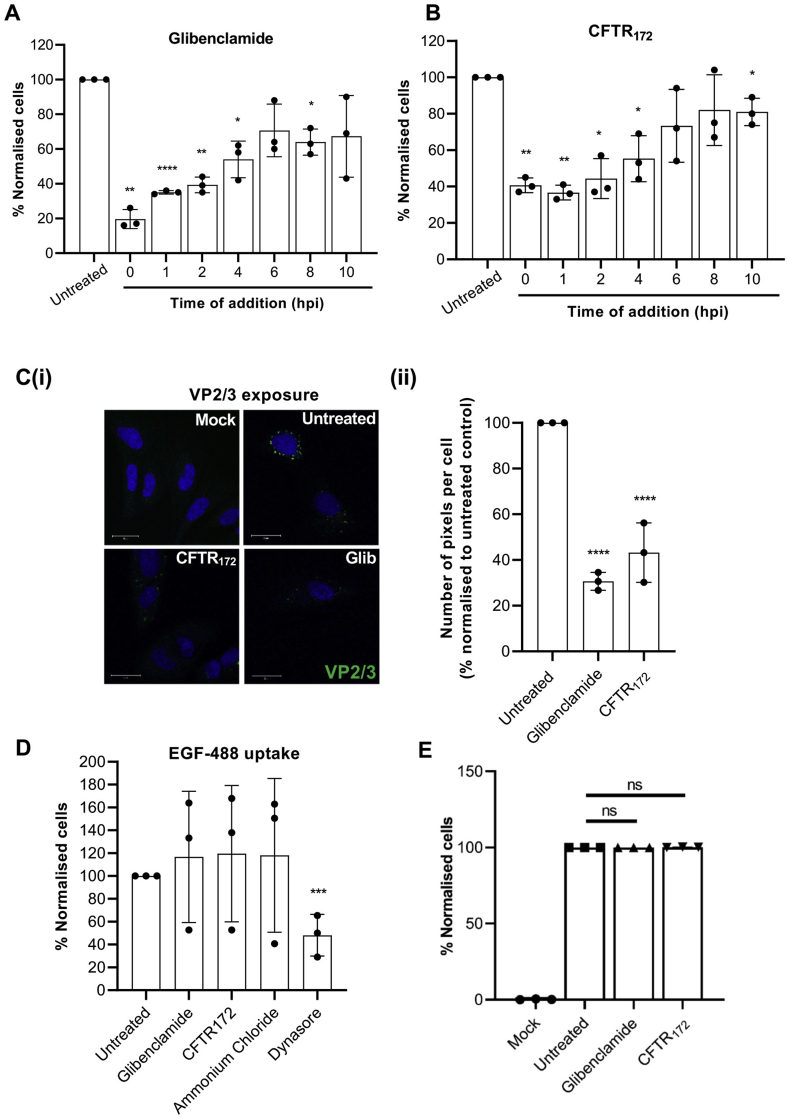
**Glibenclamide and CFTR**_**172**_**inhibit an early post-entry stage of BKPyV infection. A-B)** RPTE cells were treated with glibenclamide (20 mM) or CFTR_172_ (10 mM) at the time points indicated during a synchronous BKPyV infection. Cells were fixed and probed with anti-VP1 and anti-mouse Alexa-fluor 488 secondary antibodies. The percentage of BKPyV infected cells was quantified using IncuCyte ZOOM software and normalised to untreated cells. **Ci)** RPTE cells were treated with CFTR_172_ (10 μM) or glibenclamide (20 μM) and infected with BKPyV at an MOI of 3 for 10 h prior to fixing and staining for VP2/VP3 (green). Nuclei were stained with DAPI (blue). **Cii)** Quantification of the images from N = 3 samples. Values were normalised to untreated controls. **D)** RPTE cells were pre-treated with CFTR_172_ (10 μM), glibenclamide (20 μM) ammonium chloride (6 mM) and the dynamin inhibitor dynasore (10 μM) and pulsed with Alexa fluor-488 conjugated EGF for 45 min. Cells were fixed and the number of EGF-positive fluorescent puncta were measured using IncuCyte ZOOM software. Data are the mean ± SD; data in Fig. 4A and 4B were compared using Welch's test, and the remaining data were compared using a two-tailed unpaired *t-*test (*P < 0.05, **P < 0.005, ***P < 0.0005, ****P < 0.0001). **(E)** Purified BKPyV was labelled using the Molecular Probes Alexa Fluor® 488 Protein Labelling Kit (Invitrogen™). RPTE cells were treated with inhibitors for 24 h and cells were re-suspended in ice-cold Opti-MEM. Cells were chilled at 4 °C for 15 min prior to addition of Alexa Fluor 488-labelled BKPyV (AF488-BKPyV) diluted in Opti-MEM at MOI 0.5. The mixture was chilled at 4 °C for 1 h and unbound virus was removed through two PBS washes. Cells were fixed with 4% paraformaldehyde and virus-binding to cells was assessed using the CytoFLEX S Flow Cytometer (N = 3).

The stage of the BKPyV lifecycle that requires CFTR activity was next investigated through time-of-addition experiments using glibenclamide and CFTR_172_. Here, cells were infected for 60 min and drugs were added at defined time points post-infection (hpi). When drugs were added 10 hpi, VP1 expression was largely unaffected, suggesting that neither glibenclamide nor CFTR_172_ affect BKPyV once virus infection has been established (Fig. 4A–B). To further define the temporal window during which CFTR inhibition acts to impair BKPyV, RPTE cells were treated with inhibitor compounds at intervals between 1 and 10 hpi. Maximal impairment of BKPyV infection by CFTR inhibition was achieved within 4 hpi, and the levels of inhibition gradually decreased when drugs were added at later times (Fig. 4A–B). Taken together, these data demonstrate that BKPyV infection is primarily restricted by synchronous or early treatment with CFTR blockers (≤4 hpi) as opposed to when virus gene expression has been initiated (≥10 hpi).

The kinetics of inhibition indicated that CFTR may be required prior to delivery of BKPyV virions into the endoplasmic reticulum (ER), which typically occurs between 6 and 12 hpi (Jiang et al., 2009). To determine if glibenclamide or CFTR_172_ treatment impaired BKPyV ER-transit, we made use of the observation that upon trafficking to the ER, polyomaviruses interact with host chaperones to expose the previously obscured VP2/VP3 minor capsid proteins (Hurdiss et al., 2016; Goodwin et al., 2011; Nelson et al., 2013). Exposure of these minor capsid proteins therefore serves as a surrogate marker to assess BKPyV entry into the ER. To investigate the effects of CFTR inhibition on these processes, RPTE cells were incubated with inhibitor compounds and infected with virus for 10 h prior to fixation and immunostaining with anti-VP2/VP3 antibodies. In control cells, VP2/VP3 puncta were visible in the perinuclear region of infected cells as would be expected as this timepoint is too early for entry into the nucleus for virus replication (Fig. 4Ci). In contrast, treatment of cells with either glibenclamide or CFTR_172_ led to a reduction in the number of VP2/VP3 puncta (Fig. 4Ci-ii). Glibenclamide and CFTR_172_ had no effects on the internalisation of fluorescently labelled epidermal growth factor (EGF) (Fig. 4D), demonstrating that inhibitor treatment did not lead to a global inhibition of kidney cell uptake pathways. The EGF assays were validated by the inclusion of ammonium chloride (an inhibitor of endosomal acidification) that as expected, had no influence on EGF uptake into cells but would be predicted to inhibit post-entry EGFR trafficking. In contrast, treatment with the dynamin inhibitor, Dynasore, significantly reduced EGF internalisation. Finally we confirmed the post-entry effect of CFTR inhibition on BKPyV since neither glibenclamide nor CFTR_172_ affected the ability of BKPyV to bind to RPTECs (Fig. 4E). The culmination of these data suggest that the blockade of CFTR specifically reduces BKPyV trafficking to the ER, preventing the structural transitions necessary for exposure of the minor capsid proteins, explaining its inhibitory effects during the early stages of BKPyV infection.

## 3 Discussion

There is a pressing clinical need for therapeutics to control BKPyV infection in immunosuppressed patients. Uncontrolled BKPyV replication is responsible for PVAN and uteric stenosis in kidney transplant patients, and PVHC in haematopoietic stem cell transplant recipients. In all these patients, high viraemia is associated with an increased risk of disease progression. As such, understanding the BKPyV lifecycle is critical to the development of anti- BKPyV therapeutics that can reduce viral replication in immunosuppressed individuals, without the use of current nephrotoxic, broad spectrum anti-viral agents such as cidofovir.

In this study, we demonstrate that the sulphonylurea drug glibenclamide inhibits BKPyV infection in primary RPTE cells. The drug is most effective within the first 4 h of infection, indicating that it primarily functions at a stage of the BKPyV lifecycle prior to nuclear entry and viral transcription. Whilst considerable gaps in our understanding of these early stages of the BKPyV lifecycle remain, it is known that polyomaviruses take advantage of retrograde pathways and navigate the endolysosomal system bound to their host ganglioside receptor molecules (Nelson et al., 2013; Qian et al., 2009; Zhao and Imperiale, 2017). Endosome acidification activates a ganglioside-mediated sorting process essential for trafficking its cargo into the ER (Qian et al., 2009). Within the ER, interactions with cellular chaperones permit key conformational changes in the virus capsid that drive virus uncoating prior to nuclear entry (Walczak et al., 2014). Thus, we postulated that the cellular target for glibenclamide was a factor resident in the endosomal system. The most likely targets of glibenclamide were the sulphonylurea (SUR) ABC-transporter proteins associated with the inward rectifying K^+^ channels K_IR_ 6.1 and 6.2, which form the well-characterised K_ATP_ channels. However, a number of lines of evidence cast doubt on this assumption. Firstly, neither the silencing of SUR1, SUR2A nor SUR2B proteins influenced BKPyV infection (Aguilar-Bryan et al., 1995; Inagaki et al., 1996). Moreover, the use of additional K_ATP_ channel inhibitor compounds failed to prevent BKPyV infection. K_ATP_ channels were therefore discounted as the glibenclamide target.

CFTR is similarly sensitive to glibenclamide and is highly expressed in the proximal tubules of the kidney. The inhibitory effects of the CFTR-specific inhibitor CFTR_172_ were similar to those of glibenclamide, suggesting an overlapping target. Depletion experiments using CFTR siRNA were less efficacious at reducing VP1 expression; however, this is likely due to the limited CFTR knockdown achieved due to the known stability of the CFTR protein (half-life of over 48 h). Interestingly, the level of reduction was similar to that observed in a recent whole genome siRNA screen to identify host factors required for BKPyV infection (Zhao and Imperiale, 2017) and we consistently observed an increase in the levels of CFTR transcript in BKPyV infected cells. It is plausible that the virus may actively drive CFTR expression if the host factor is essential for the virus lifecycle. Alternatively, a redundant Cl^-^ transport channel in human kidney cells that is also targeted by both CFTR_172_ and glibenclamide cannot be completely discounted.

A number of studies indicate a role for CFTR in endosome trafficking and fusion (Bradbury, 1999). In particular, CFTR is enriched in the apical endosomes of proximal tubule cells (Morales et al., 1996) and co-localises with the vacuolar ATPase (v-ATPase), which is essential for endosome acidification (Collaco et al., 2013). Given that a parallel Cl^−^ conductance into the interior-positive membrane potential created by v-ATPase is essential for endosome acidification, it is possible that CFTR regulates these processes in kidney cells (Carraro-Lacroix et al., 2010). Whilst we have not yet addressed this directly, we did observe that treatment with glibenclamide or CFTR_172_ significantly reduced the transport of virions to the ER, a step that is blocked when endosome acidification is impaired (Jiang et al., 2009; Qian et al., 2009).

Specific BKPyV genotypes are predictive of the clinical outcome of infection (Pastrana et al., 2013; Solis et al., 2018; Korth et al., 2019). In addition to BKPyV, other polyomaviruses cause disease in humans including JC polyomavirus and Merkel cell polyomavirus, the former causing PVAN in select cases (DeCaprio and Garcea, 2013). Since these viruses also take advantage of retrograde trafficking, it is likely that CFTR inhibitors will be similarly active against these pathogens. Two other potential polyomaviruses (LIPyV and QPyV) have also been detected in human specimens and their susceptibility to CFTR modulation may further highlight a pan-family dependence (Gheit et al., 2017; Ondov et al., 2019). Targeting host components has the distinct advantage of avoiding the evolution of virus escape mutants. Indeed, as glibenclamide is currently in clinical use, our work suggests it may represent a new and safe drug for the treatment of polyomavirus disease. A caveat is that glibenclamide must be administered to patients prior to-, or early during infection (≤4 hpi). However, given the limited side-effects of this compound, its administration to vulnerable patients could prevent future BKPyV infections and/or reduce viral spread. As such, the targeting of essential host channels such as CFTR may be a promising avenue of research to treat BKPyV infection in immunosuppressed individuals.

## 4 Experimental procedures

### 4.1 Cell culture

RPTE cells (Lonza) were maintained in Renal Epithelial Cell Growth Medium 2 with supplements (ready-to-use) (PromoCell) at 37 °C in a 5% CO_2_ humidified incubator. Cells were maintained up to passage 7 (Panou et al., 2018).

### 4.2 BKPyV infections

RPTE cells seeded into 6-well dishes (2 x 10^5^ cell/ml) were chilled at 4 °C for 15 min and infected with BKPyV (Dunlop) stocks at the indicated MOIs in serum free media (Opti-MEM) at 4 °C. After 2 h, cells were incubated at 37 °C to allow virus infection to proceed.

### 4.3 Ion channel modulators

Stock solutions of TEA, BaCl_2_, 4-AP, 5-HD and U-373883A (Sigma Aldrich) were produced in dH_2_O. Quinidine, glibenclamide, tolbutamide, VU-591, Dynasore (dynamin inhibitor) and CFTR_172_ were produced in DMSO.

### 4.4 Virus drug assays

RPTE cells (2 x 10^3^ cells per well) in 96-well plates were chilled at 4 °C for 15 min and infected with BKPyV in Opti-MEM (MOI 0.5) for 2 h at 4 °C. Cells were then washed to remove unbound virus and treated with the indicated ion channel compounds. After 48 h, cells were fixed, stained and processed for IncuCyte ZOOM analysis.

### 4.5 Time of addition experiments

RPTE cells (2 x 10 ^3^ cells per well) in 96-well plates were chilled at 4 °C for 15 min and infected with BKPyV in Opti-MEM (MOI 0.5) for 2 h at 4 °C. Virus was then removed, cells were washed in PBS, and treated with the indicated compounds at 0, 1, 2, 4, 6, 8 and 10 hpi. Cells were incubated for a total of 48 h. Cells were then fixed, stained and analysed by IncuCyte ZOOM analysis.

### 4.6 siRNA transfections

RPTE cells were transfected with 50 nM of pooled siRNA using lipofectamine 2000 (1:2 ratio). After 48 h, cells were infected with BKPyV (MOI of 0.5) for 2 h at 37 °C. Virus was then removed and cells were incubated in fresh media for a further 48 h prior to analysis.

### 4.7 Preparation of protein lysates

RPTE cells were lysed in E1A lysis buffer (50 mM HEPES pH 7.0, 250 mM NaCl, 0.1% NP-40, 1 mM EDTA, 1 mM DTT and 1 x Protease inhibitor cocktail EDTA-free (Jiang et al., 2009). Lysates were sonicated 3 times, incubated on ice for 1 h, and centrifuged at 13,000 rpm for 5 min.

### 4.8 Western blotting

Proteins were resolved by 10% SDS PAGE and transferred onto HyBondTM-C Extra mixed ester nitrocellulose membranes (Amersham Biosciences) using a semi-dry Turbo-Blotter (BioRad). Membranes were probed with primary antibodies against BKPyV VP1 (PAb597: 1:250) an anti-SV40-VP1 antibody, GAPDH (Santa Cruz sc-47724: 1:5000) and BKPyV VP2/VP3 (Abcam ab53983: 1:1000) overnight at 4 °C as previously described (Panou et al., 2018).

### 4.9 Quantitative real-time PCR

Total RNA was extracted using the E.Z.N.A. Total RNA Kit I (Omega Bio- Tek) according to the manufacturer's protocol. RNA (1 μg) was DNase treated using RQ1 RNase-Free DNase (Promega) and reverse transcribed with a mixture of random and oligo (dT) primers using qScriptTM cDNA SuperMix kit (Bio-Rad LaboratoriesRT-qPCRs were performed using the QuantiFast SYBR Green PCR kit (Qiagen). Data were analysed using the ^ΔΔ^Ct method on Rotor-Gene 6000 software (Panou et al., 2018). Values were normalised to *U6*). Primer pairs were as follows: VP1 F 5′ CCA GAT GAA AAC CTT AGG GGC TT 3′, VP1 R 5′ AGA TTT CCA CAG GTT AGG TCC TCA TT 3′, U6 F 5′ CTC GCT TCG GCA CA 3′, U6 R 5′ AAC GCT TCA CGA ATT TGC GT 3′, CFTR F1 5′ TGG ATC GCT CCT TTG CAA GT 3′, CFTR R1 5′ AAG TCC ACA GAA GGC AGA CG 3′, CFTR F2 5′ AGG AAC GCT CTA TCG 3′, CFTR R2 5′ TGA CAG CTT TAA AGT CTT 3'.

### 4.10 IncuCyte Zoom analysis

RPTE cells were cultured in 96-well plates at a density of 2 x 10^3^ cells/ml for 16 h prior to infection with BKPyV. Virus was then removed and cells were incubated for a further 48 h. Infected cells were then washed in PBS for 5 min and fixed in 4% paraformaldehyde for 10 min at room temperature. Cells were permeabilised in 0.1% Triton-X100 in PBS (v/v) for 15 min and blocked in 1% BSA in PBS (Sigma Aldrich) for 30 min at room temperature. Cells were then probed with primary anti-VP1 (PAb597) (1:250) antibodies overnight at 4 °C and labelled with secondary anti-mouse Alexa Fluor 488-conjugated antibodies (1:250) (Thermo Fisher Scientific) for 2 h at room temperature. Cells were washed and imaged using the IncuCyte Dual colour ZOOM FLR system (Essen Bioscience). Images were collected with single scans and fluorescent object counts per well were analysed using ZOOM software (Stewart et al., 2015).

### 4.11 Cell viability assays

RPTE cells in 96-wells were treated with inhibitor compounds for up to 48 h. MTT reagent (1 mg/ml) was added in serum-free media and cells were incubated at 37 °C in the dark for 30 min. MTT solution was then replaced with 100 μl DMSO for 5 min, and optical densities were read at 570 nm on a microplate reader.

### 4.12 Immunofluorescence staining

RPTE cells on glass coverslips were BKPyV infected for 2 h and drug treated. Cells were maintained at 37 °C in a 5% CO_2_ humidified incubator for a further 10 h. Cells were washed in PBS, fixed with 4% paraformaldehyde and permeabilised in 0.1% Triton-X100 in PBS. Cells were then blocked in 1% BSA in PBS for 30 min at room temperature and labelled with anti-VP2/VP3 (ab53983) (1:500) antibodies overnight at 4 °C. Cells were then washed and labelled with secondary anti-rabbit Alexa Fluor 488-conjugated antibodies (1:1000) (Thermo Fisher Scientific) for 2 h at room temperature. Coverslips were mounted with DAPI-containing ProLong Gold antifade reagent. Immunofluorescence analysis was performed on a LSM 880 Zeiss confocal microscope. Images were analysed for a number of pixels using Zeiss bioimaging software.

### 4.13 EGF uptake assays

RPTE cells in 12-well plates were treated with the indicated drugs for 30 min and pulsed with 2 μg/ml Alexa Fluor® 488 EGF in Opti-MEM. Cells were incubated at 37 °C for a further 20 min and fixed in 4% paraformaldehyde. EGF uptake was assessed on an IncuCyte Dual colour ZOOM FLR (Essen Bioscience). Images were collected with single scans and analysed using ZOOM software (fluorescent object counts per well).

### 4.14 Virion binding assays

Purified BKPyV was labelled using the Molecular Probes Alexa Fluor® 488 Protein Labelling Kit (Invitrogen™) according to the manufacturers protocols. RPTE cells were treated with inhibitors for 24 h prior to detachment, and cells were re-suspended in ice-cold Opti-MEM. Cells were chilled at 4 °C for 15 min prior to addition of Alexa Fluor 488-labelled BKPyV (AF488-BKPyV) diluted in Opti-MEM at MOI 0.5. The mixture was chilled at 4 °C for 1 h and unbound virus was removed through 2 x PBS washes. Cells were fixed with 4% paraformaldehyde and virus-binding was assessed using the CytoFLEX S Flow Cytometer (Beckman Coulter).

## Declaration of competing interest

The authors declare that they have no conflicts of interest with the contents of this article.
